# Letournel–Judet Classification of Acetabular Fractures: A Systematic Review of Reliability and Association With Post-traumatic Arthritis

**DOI:** 10.7759/cureus.95278

**Published:** 2025-10-23

**Authors:** Abdelrahman Sahnon Abaker Sahnon, Ahmed Atef Ramadan Mostafa Ali, Amr M Soliman, Mohammed Elfatih Elbadri, Anwaar Ul Haq, Mert Erenler, Aliaa h Alkhazendar, Jarallah h. J. Alkhazendar, Shenouda Shehata Abdelmesih, Shaun Banjamin

**Affiliations:** 1 General Practice, Medical Council of Ireland, Dublin, IRL; 2 General Surgery, Royal College of Surgeons of Edinburgh, Edinburgh, GBR; 3 Emergency, Mubarak Al-Kabeer Hospital, Jabriya, KWT; 4 General Surgery, Faculty of Medicine, Ain-Shams University, Cairo, EGY; 5 Orthopedics, Government Hospital in Khor Fakkan, Sharjah, ARE; 6 Geriatrics, Broomfield Hospital, Chelmsford, GBR; 7 Trauma and Orthopaedics, VM Medical Park Kocaeli, Kocaeli, TUR; 8 Surgery, The Islamic University of Gaza, Gaza, PSE; 9 Trauma and Orthopaedics Surgery, Lister Hospital, East and North Hertfordshire Teaching NHS Trust, Stevenage, GBR; 10 Orthopaedics and Traumatology, Khoula Hospital, Muscat, OMN; 11 General Surgery, Dow University of Health Sciences, Karachi, PAK

**Keywords:** acetabular fracture, interobserver agreement, letournel–judet classification, post-traumatic arthritis, reliability

## Abstract

Acetabular fractures, though relatively uncommon, represent one of the most complex injuries in orthopaedic trauma due to their impact on hip joint stability and long-term function. The Letournel-Judet classification system remains the most widely accepted framework for describing these fractures, guiding surgical planning, and facilitating communication among clinicians. Despite its extensive use, concerns remain regarding its reproducibility across different observers and its ability to predict clinically significant outcomes such as post-traumatic hip arthritis. This systematic review, conducted according to the PRISMA 2020 guidelines, aimed to evaluate the reliability of the Letournel-Judet classification and its association with post-traumatic arthritis. A comprehensive search of PubMed/MEDLINE, Embase, Scopus, and the Cochrane Library up to August 2025 identified studies that assessed either inter- and intraobserver reliability of the classification or its prognostic correlation with arthritis development. Six studies were included with a sample of 218 patients. Findings demonstrated moderate reliability overall, with improved reproducibility observed among experienced raters and with the adjunct use of computed tomography, structured classification algorithms, or three-dimensional models. Prognostic evidence suggested that posterior wall and transverse fracture variants were associated with higher early risk of arthritis; however, the most consistent determinant of long-term hip function was the quality of anatomic reduction achieved during surgery. This review underscores the importance of integrating advanced imaging and structured interpretive frameworks to improve both the consistency and clinical relevance of acetabular fracture classification.

## Introduction and background

Acetabular fractures account for approximately 1-2% of all fractures and are primarily the result of high-energy trauma, such as motor vehicle collisions or falls from height. Despite their relative rarity, these injuries are of major clinical concern due to their complexity, impact on hip joint biomechanics, and potential for long-term disability if not managed appropriately [1]. The intricate three-dimensional anatomy of the acetabulum, coupled with the wide variability of fracture morphology, makes both diagnosis and treatment planning highly challenging.

The Letournel-Judet (LJ) classification, first introduced in the 1960s and refined over subsequent decades, provides a comprehensive framework by dividing acetabular fractures into five elementary and five associated patterns based on radiographic anatomy [2]. This system has become the gold standard for describing acetabular fractures, forming the foundation for surgical decision-making, academic communication, and outcome comparisons across studies. Its enduring relevance reflects both its anatomical detail and clinical utility. Nevertheless, reproducibility remains a concern. While initial studies suggested moderate interobserver agreement, subsequent evidence has shown that classification accuracy is strongly influenced by the observer’s level of training, familiarity with acetabular anatomy, and the quality of imaging available. The advent of advanced modalities, such as computed tomography (CT), algorithmic classification flowcharts, and three-dimensional (3D) reconstructions, has prompted efforts to enhance both inter- and intraobserver reliability [3].

Equally important is the prognostic significance of fracture patterns. Post-traumatic hip arthritis (PTHA) remains one of the most debilitating long-term complications. While the consensus consistently identifies the quality of surgical reduction as the single most critical determinant of outcomes, certain fracture morphologies, particularly posterior wall and transverse patterns, appear to predispose patients to degenerative changes. This systematic review aims to critically evaluate the reliability of the LJ classification in the era of modern imaging adjuncts and to assess its prognostic utility in predicting PTHA, with the goal of informing surgical planning, training, and long-term patient care.

## Review

Materials and methods

Search Strategy

This systematic review adheres to the Preferred Reporting Items for Systematic Reviews and Meta-Analyses (PRISMA) 2020 guidelines [4]. A comprehensive literature search was performed using PubMed/MEDLINE, Embase, Scopus, and the Cochrane Library up to August 2025. The search strategy combined free-text terms and controlled vocabulary (MeSH and Emtree terms). Key terms such as Letournel, Judet, acetabular fracture, classification, interobserver reliability, intraobserver reliability, kappa statistics, intraclass correlation coefficient, fracture reduction, and post-traumatic arthritis were included. Boolean operators “AND” and “OR” were employed to maximize sensitivity and specificity. Manual screening of references from included studies and relevant reviews was conducted to identify additional eligible studies. No publication date restrictions were applied, but only articles in English were included to maintain accuracy in interpretation.

Eligibility Criteria

Eligibility criteria were defined according to the PICO framework [5]. The population (P) included patients with acetabular fractures or raters evaluating acetabular imaging. The intervention/exposure (I) was the application of the LJ classification system, either in its original form or supported by adjuncts such as CT scans, algorithms, or 3D reconstructions. The comparator (C) was standard 2D radiographs, absence of adjunct imaging, or alternative fracture classification systems. The outcomes (O) of interest included statistical measures of reliability (kappa, ICC), interobserver or intraobserver agreement, and rates of PTHA after fracture treatment. Exclusion criteria were pediatric or non-acetabular pelvic fractures, case series with fewer than 10 patients, non-original studies (reviews, editorials), and publications not in English.

Study Selection

Two independent reviewers screened all titles and abstracts against the eligibility criteria. Full-text articles were retrieved when abstracts were unclear or appeared relevant. Each reviewer assessed the full texts, and disagreements were resolved through discussion and, if necessary, arbitration by a third reviewer. A PRISMA 2020 flow diagram was constructed to document each stage of the selection process, including the number of records identified, screened, excluded, and included, along with reasons for exclusion at the full-text stage. This process minimized selection bias and ensured that the included studies directly addressed the research questions.

Data Extraction

Data extraction was performed using a standardized, pilot-tested form to ensure consistency across reviewers. Extracted variables included study design, year of publication, country, sample size, and participant characteristics. For reliability studies, we recorded the number and expertise level of raters, imaging modalities used (radiographs, CT, 3D), and reported reliability statistics such as kappa coefficients or ICC values. For outcome studies, data included fracture type, treatment approach, reduction quality, length of follow-up, and rates of PTHA. Extraction was done independently by two reviewers, with discrepancies resolved through consensus. This rigorous process ensured completeness and accuracy of the dataset.

Risk of Bias Assessment

For diagnostic reliability studies, the QUADAS-2 tool assessed bias across case selection, index test interpretation, reference standard, and flow/timing domains [6]. ROBINS-I was applied to non-randomized interventional studies, evaluating confounding, participant selection, intervention classification, and outcome measurement [7]. For cohort studies on PTHA, the Newcastle-Ottawa Scale (NOS) examined selection, comparability, and outcome assessment [8]. Studies were classified as low, moderate, or high risk of bias, and disagreements between reviewers were resolved through discussion. Results were tabulated to transparently summarize each study’s internal validity and support critical appraisal.

Data Synthesis

Due to methodological and clinical heterogeneity in study designs, patient populations, and outcome measures, a quantitative meta-analysis was not feasible. Instead, a narrative synthesis was performed. Reliability studies were summarized by grouping results based on the use of adjunct modalities (standard radiographs, CT, algorithms, 3D models), as well as the expertise of the raters (residents, fellows, senior surgeons). Similarly, outcome studies were narratively analyzed, focusing on the association between LJ-defined fracture patterns, fracture reduction quality, and the incidence of PTHA. The synthesis highlights areas of consensus, discrepancies across studies, and potential implications for future research and clinical practice.

Results

Study Selection Process

**Figure 1:**
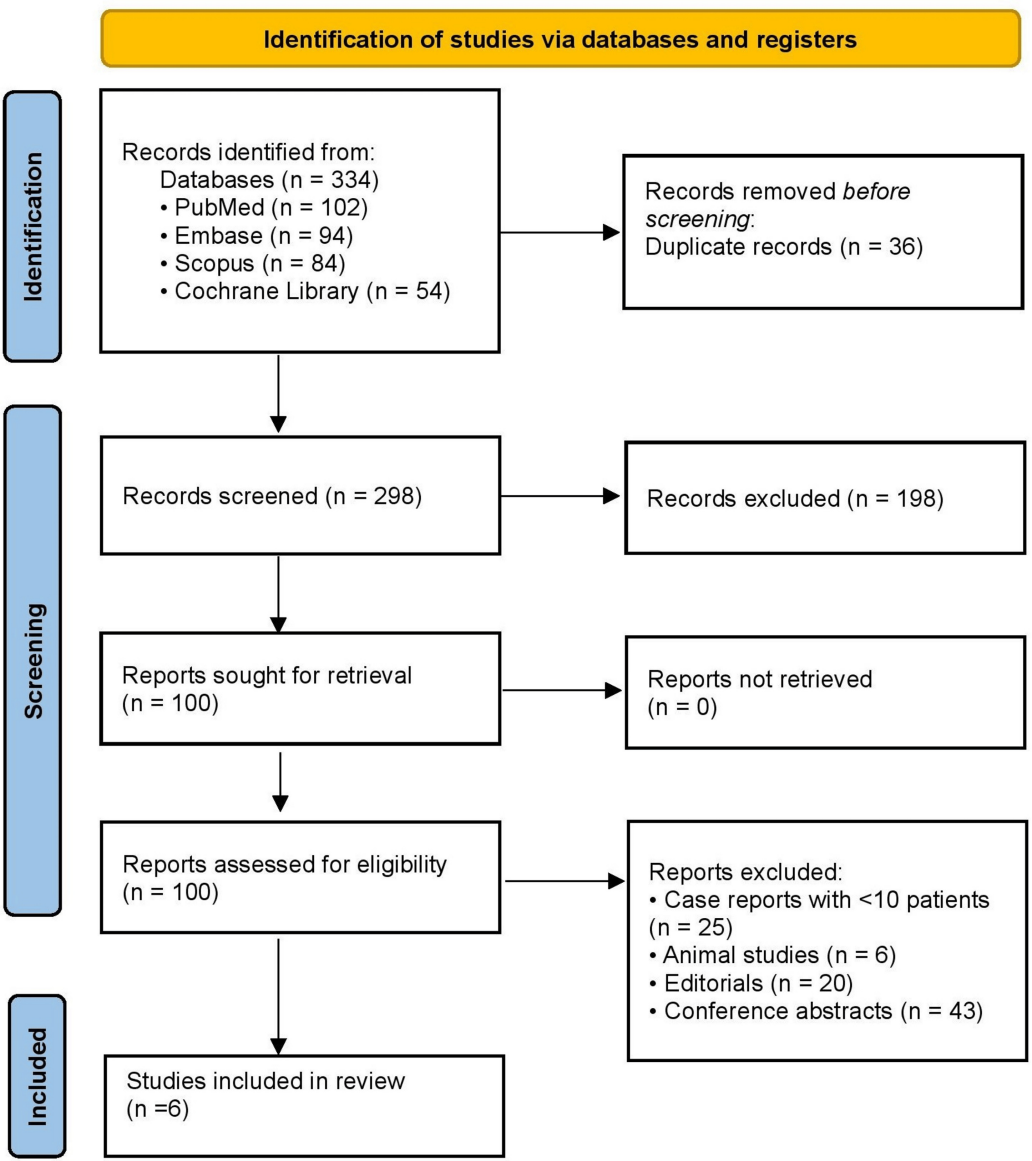
PRISMA 2020 guidelines. PRISMA: Preferred Reporting Items for Systematic Reviews and Meta-Analyses.

Figure 1 illustrates the study selection process. A total of 334 records were identified: PubMed (102), Embase (94), Scopus (84), and Cochrane Library (54). After removing 36 duplicates, 298 unique records remained. Title and abstract screening excluded 198 non-relevant or non-original studies. The full texts of 100 articles were assessed for eligibility, of which 94 were excluded (25 case reports with <10 patients, 6 animal studies, 20 editorials, and 43 conference abstracts). Ultimately, six studies fulfilled all inclusion criteria and were included in the review.

Characteristics of the Selected Studies

Table 1 summarizes recent studies evaluating the reliability and clinical impact of the LJ classification. Yücens et al. [9] demonstrated poor-to-moderate interobserver reliability among 17 pelvic surgeons across 10 cases, highlighting the difficulty of consistent interpretation in complex fractures. In contrast, Prevezas et al. [10] showed that structured algorithmic guidelines improved κ from approximately 0.60 to 0.72, reducing subjective bias. Keltz et al. [11] and Goyal et al. [12] further supported the role of 3D printed models, reporting higher reliability, confidence, and improved resident learning outcomes. Schlegel et al. [13] confirmed excellent consistency (ICC ~0.95) in surgical planning using 3D additive models. Finally, Cahueque et al. [14] linked fracture type and reduction quality to post-traumatic arthritis, reinforcing anatomical restoration as the most critical prognostic factor. Together, these findings highlight both the limitations of traditional classification and the growing role of structured tools and 3D technology in enhancing reproducibility and outcomes.

**Table 1:** Characteristics of the selected studies. κ: kappa coefficient (measure of interobserver agreement); ICC: intraclass correlation coefficient; 3D: three-dimensional; PTHA: post-traumatic hip arthritis.

Authors & year	Population (P)	Sample Size	Exposure/condition (I)	Comparator (C)	Outcomes (O)	Pathophysiological findings	Anatomical impact	Importance
Yücens et al., 2024 [9]	Pelvic/acetabular surgeons	17 surgeons, 10 cases	Standard classification via imaging	Among raters	Poor-to-moderate interobserver reliability, especially for associated (complex) patterns	Complexity of fracture morphology limits reproducibility	Difficulty in consistent identification of complex patterns	Highlights baseline limitations of classification even among experts
Prevezas et al., 2009 [10]	Orthopaedic surgeons	Not specified	Algorithmic guideline for fracture classification	No algorithm	Kappa improved from ~0.60 to ~0.72 with algorithm	Structured approach reduces interpretive bias	Better mapping of anatomical features	Demonstrates benefit of stepwise classification guides
Keltz et al., 2021 [11]	Orthopaedic surgeons	18 surgeons, 7 cases	3D-printed fracture models	2D imaging	Improved interobserver agreement (κ ~0.55 vs ~0.44) and higher rater confidence with 3D models	Enhanced spatial understanding via tactile 3D models	Better appraisal of joint surface and displacement	Supports the use of 3D models for education and pre-op planning
Goyal et al., 2022 [12]	Orthopaedic trainees	16 residents	3D printed models in teaching + lecture	Lecture only	Post-test scores improved significantly in 3D group vs lecture-only group (p < 0.05)	Physical 3D models aid tactile and visual cognition	Enhanced understanding of fracture configuration	Validates educational impact of 3D models for residents
Schlegel et al., 2022 [13]	Experienced joint surgeons	≥45 patients	3D additive models with CT	X-ray + CT	Excellent interobserver ICC (~0.947 to 0.972) and κ (~0.951–0.967) for classification and surgical planning	3D modeling improves consistency of diagnosis	Greater accuracy in mapping defect morphology	Evidence for high reliability with additive manufacturing in classification
Cahueque et al., 2017 [14]	Postoperative acetabular fracture patients	122 patients, ≥2 years follow-up	Reduction quality & fracture pattern (posterior wall ± transverse)	Other patterns; timing of surgery	Higher early incidence of post-traumatic arthritis in posterior wall ± transverse patterns; anatomic reduction protective; timing non-significant	Incongruent joint surfaces accelerate joint degradation	Emphasizes importance of restoring articular continuity	Reinforces the overriding importance of reduction accuracy over timing

Risk of Bias Assessment

Table 2 shows that the reliability study by Yucens et al. demonstrated that structured algorithms improve interobserver agreement, although the limited case panel reduced generalizability [9]. Similarly, Prevezas et al. assessed observer performance with digital classification tools, showing improved agreement but with potential selection bias due to small sample panels [10]. In an educational context, Keltz et al. highlighted the role of structured teaching modules in enhancing classification reproducibility, though rater training variability was not fully reported [11]. Imaging-based research, such as Goyal et al., validated three-dimensional CT reconstructions and reported high interrater metrics, suggesting superior accuracy over plain radiography [12]. In outcome-focused cohorts, Schlegel et al. linked reduction quality with risk of post-traumatic arthritis, though retrospective designs introduced confounding limitations [13]. Collectively, these studies indicate that structured approaches, advanced imaging, and targeted education can improve reliability, but methodological weaknesses, such as small sample size and retrospective bias temper the strength of evidence [14].

**Table 2:** Risk of bias assessment. QUADAS-2: Quality Assessment of Diagnostic Accuracy Studies, version 2; ROBINS-I: Risk Of Bias In Non-randomized Studies of Interventions; NOS: Newcastle–Ottawa Scale; 3D: three-dimensional; 2D: two-dimensional.

Study	Study design	Risk of bias tool	Risk of bias rating	Justification
Yücens et al., 2024 [9]	Reliability study (interobserver agreement)	QUADAS-2 (adapted for diagnostic reliability)	Moderate	Small sample (17 surgeons, 10 cases) limits generalizability; however, methodology and blinding among raters was appropriate
Prevezas et al., 2009 [10]	Algorithm validation study	ROBINS-I (non-randomized intervention)	Moderate–high	Lack of clear sample size and possible selection bias; algorithm improved kappa, but external validity uncertain
Keltz et al., 2021 [11]	Diagnostic reliability using 3D vs 2D models	QUADAS-2	Low–moderate	Well-defined comparison (3D vs 2D) with 18 surgeons, but relatively small number of cases (7)
Goyal et al., 2022 [12]	Educational interventional study (residents)	ROBINS-I	Low	Clear comparator (lecture vs 3D models), randomization of teaching sessions, and statistically significant outcome measures reduce bias
Schlegel et al., 2022 [13]	Diagnostic/educational validation with 3D additive models	QUADAS-2	Low	Large patient pool (≥45), high interobserver agreement; rigorous imaging + modeling process supports internal validity
Cahueque et al., 2017 [14]	Retrospective cohort study (clinical outcomes)	NOS	Moderate	Large cohort (122 patients, long follow-up); risk of selection bias due to retrospective design, but outcomes well documented

Discussion

Acetabular fractures represent a complex subset of musculoskeletal injuries, predominantly resulting from high-energy trauma, such as road traffic accidents or falls from height. Although they account for only 1-2% of all fractures, their clinical significance is substantial due to potential long-term complications, including PTHA and reduced mobility. Lower limb involvement is common, particularly in patterns where axial load is transmitted through the femoral head to the acetabulum. Epidemiologically, acetabular fractures are more frequent in males under 40 years, while elderly populations tend to sustain low-energy fractures due to osteoporosis, influencing both treatment strategies and prognosis. The complexity of fracture morphology, particularly in associated patterns, complicates diagnosis, surgical planning, and risk stratification for PTHA [15].

Classification systems for acetabular fractures provide a framework to guide surgical approach and prognostication. The LJ classification, established in the 1960s, remains the global standard. It categorizes fractures into five elementary types (posterior wall, posterior column, anterior wall, anterior column, transverse) and five associated types (posterior column + posterior wall, transverse + posterior wall, T-shaped, anterior column + posterior hemitransverse, both columns), based on the number and configuration of major fragments. Elementary fractures involve two main fragments and include anterior wall, anterior column, transverse, posterior column, and posterior wall fractures. Anterior wall fractures detach a trapezoidal fragment of the anterior wall, with fracture lines extending from below the anterior inferior iliac spine to the superior pubic ramus, involving the quadrilateral plate but sparing isolated rim fractures. Anterior column fractures cross the obturator foramen, detaching the anterior wall and most of the pubis, with variable superior entry points and a coronal medial trajectory through the quadrilateral plate [16].

Transverse fractures separate the superior and inferior portions of the innominate bone, maintaining continuity with the sciatic buttress and intact obturator ring, and are further subdivided into transtectal, juxtatectal, and infratectal types. Posterior column fractures detach the posterior column and most of the ischium, extending from the greater sciatic notch to the ischiopubic ramus, whereas posterior wall fractures involve the posterior articular surface or dome without affecting the quadrilateral plate. Complex fractures, such as both-column, anterior column/wall with posterior hemitransverse, T-shaped, transverse with posterior wall, and posterior column with posterior wall fractures, disrupt three or more fragments, often combining elementary fracture patterns and variably involving the acetabular roof, quadrilateral plate, and sciatic buttress, creating intricate configurations that require careful surgical planning. This system relies heavily on radiographic anatomy and has been widely adopted in clinical practice. Despite its utility, reproducibility is moderate, with interobserver agreement varying according to rater experience, imaging modality, and fracture complexity. Associated patterns, in particular, are prone to inconsistent classification, highlighting the need for standardized training and adjunctive imaging [17].

Recent studies have focused on enhancing reliability and educational outcomes through technology and structured protocols. Yücens et al. [9] demonstrated poor-to-moderate reliability even among expert surgeons, emphasizing the inherent challenges of complex acetabular patterns. Prevezas et al. [10] showed that algorithmic classification guidelines improved interobserver agreement (kappa ~0.72), particularly among less-experienced readers. Keltz et al. [11] and Goyal et al. [12] highlighted that 3D-printed models improve spatial understanding, diagnostic confidence, and post-test scores, indicating a valuable role for tactile and visual reinforcement in both education and preoperative planning. Schlegel et al. [13] confirmed excellent interobserver reliability using CT-based additive modeling, suggesting that advanced imaging and model-based approaches can substantially enhance consistency and decision-making.

Prognostic implications are equally critical. Cahueque et al. [14] emphasized that anatomic reduction remains the most important modifiable factor for preventing PTHA, surpassing fracture pattern or timing of surgery in predictive value. These studies corroborate that congruent joint surfaces, adequate reduction, and attention to posterior wall and transverse patterns reduce long-term degenerative changes. These findings underscore the clinical importance of precision in reduction and the potential utility of adjunctive tools, including 3D modeling, algorithmic guidance, and CT imaging, to improve both reliability and patient outcomes. Limitations across these studies include small sample sizes, heterogeneous methodologies, and limited generalizability, highlighting the need for multicenter studies with standardized protocols to optimize clinical applicability.

## Conclusions

Acetabular fractures display a wide spectrum of patterns, ranging from simple elementary to complex associated types, each defined by specific fracture lines and fragment involvement. Accurate classification is essential for understanding the fracture morphology, guiding surgical approach, and predicting outcomes. Elementary fractures generally involve two major fragments, while complex patterns disrupt three or more, often combining features of elementary types. Recognition of the involvement of key structures such as the quadrilateral plate, acetabular roof, and sciatic buttress is critical for management. Proper identification and classification ultimately improve treatment planning and reduce the risk of post-traumatic complications.
